# The lived experiences of chronic pain among immigrant Indian-Canadian women: A phenomenological analysis

**DOI:** 10.1080/24740527.2020.1768835

**Published:** 2020-09-24

**Authors:** Nida Mustafa, Gillian Einstein, Margaret MacNeill, Judy Watt-Watson

**Affiliations:** a Dalla Lana School of Public Health, University of Toronto, Toronto, Ontario, Canada; b Department of Psychology, University of Toronto, Toronto, Ontario, Canada; c Department of Gender Studies, Linköping University, Linköping, Sweden; d Graduate Department of Exercise Sciences, University of Toronto, Toronto, Ontario, Canada; e Lawrence S. Bloomberg Faculty of Nursing, Toronto, Ontario, Canada

**Keywords:** chronic pain, Indian women, immigration, sociocultural context and pain

## Abstract

**Background**: Chronic pain is a growing public health concern affecting 1.5 million people in Canada. In particular, it is a concern among the expanding immigrant population, because immigrant groups report higher pain intensity than non-immigrants. In 2011, the Indian population became the largest visible minority group and continues to be the fastest growing. Though the prevalence of chronic pain among Canadian Indians is unknown, research has found a higher prevalence among Indian women than men in India, Malaysia, Singapore, and the United Kingdom, with women reporting more severe pain. An understanding of how pain is experienced by this particular group is therefore important for providing culturally sensitive care.

**Aims:**

This study explores the lived experiences of chronic pain among immigrant Indian women in Canada.

**Methods:**

Thirteen immigrant Indian women participated in one-on-one interviews exploring daily experiences of chronic pain.

**Results:**

Using thematic analysis informed by van Manen’s phenomenology of practice, four themes emerged: (1) the body in pain, (2) pain in the context of lived and felt space, (3) pain and relationships, and (4) pain and time. Women revealed that their experiences were shaped by gender roles and expectations enforced through culture. Specifically, a dual gender role was identified after immigration, in which women had to balance traditional household responsibilities of family labor and care alongside employment outside the home, exacerbating pain.

**Conclusions:**

This research uncovers the multifaceted nature of chronic pain and identifies factors within the sociocultural context that may place particular groups of women at greater risk of living with pain.

## RÉSUMÉ

**Contexte**: La douleur chronique est un problème de santé publique croissant qui touche 1,5 million de personnes au Canada. Elle est particulièrement préoccupante au sein de la population immigrante en expansion, car les groupes d’immigrants signalent une intensité de douleur plus élevée que les non-immigrants. En 2011, la population indienne est devenue le plus grand groupe de minorités visibles et continue d’être celui qui connaît la croissance la plus rapide. Bien que la prévalence de la douleur chronique chez les Indo-Canadiens soit inconnue, des études ont montré que la prévalence de cette maladie chez les femmes indiennes était supérieure à celle des hommes en Inde, en Malaisie, à Singapour et au Royaume-Uni, les femmes faisant état de douleurs plus intenses. Une compréhension de la manière dont la douleur est ressentie par ce groupe particulier est donc importante pour fournir des soins adaptés à la culture.

**Objectifs**: Cette étude se penche sur les expériences de douleur chronique vécues par les immigrantes indiennes au Canada.

**Méthodes**: Treize immigrantes indiennes ont participé à des entretiens individuels portant sur leurs expériences de douleur chronique quotidiennes.

**Résultats**: Grâce à une analyse thématique fondée sur la phénoménologie de la pratique de van Manen, quatre thèmes ont émergé : (1) le corps douloureux, (2) la douleur dans le contexte de l’espace vécu et ressenti, (3) la douleur et les relations, et (4) la douleur et le temps. Les femmes ont révélé que leur expérience était façonnée par les rôles sexo-spécifiques et les attentes étaient imposées par la culture. Plus précisément, un double rôle sexo-spécifique a été découvert après l’immigration, dans laquelle les femmes doivent équilibrer les responsabilités traditionnelles du travail familial et des soins parallèlement à l’emploi en dehors du domicile, ce qui exacerbe la douleur.

**Conclusions**: Cette étude révèle la nature multidimensionnelle de la douleur chronique et recense les facteurs propres au contexte socioculturel qui peuvent placer des groupes particuliers de femmes à plus grand risque de vivre avec la douleur.

## Introduction

Chronic pain is defined as persistent pain that lasts longer than the normal time of healing^1^ and is associated with prolonged physical and psychological impairment.^2,3^ In Canada, chronic pain is a public health concern involving an estimated 1.5 million people nationwide. It is a growing concern among immigrant communities, as Canada has a large and growing number of immigrants, who make up approximately 22% of the total population.^4^ In 2011, the Indian population became the largest visible minority group and is the fastest growing community in Canada.^5^ Based on quantitative studies, more Indian women than men report constant and severe pain in both India and the diaspora.^6^ Thus, an understanding of how chronic pain is experienced by Indian women, especially in Canada, is necessary and will contribute to knowledge-based and culturally sensitive care.^7^

### Culture and Pain

Considerable research has examined pain from a biological perspective; however, studying the influence of culture—beliefs and practices—is a relatively new and growing area.^8–10^ *Culture* is defined as “the patterned ways that humans have learned to think about and act in their world; it involves learned, shared styles of thought and behavior which replicate the social structure of their world.”^11(p15)^ Culture is also a significant factor in shaping pain communication, beliefs, and coping behaviors,^12^ as well as perceptions of pain.^10^ Therefore, in order to better understand pain experiences among diverse populations, an exploration of culture in shaping these experiences is paramount.

With respect to Indian culture in particular, research has shown that cultural norms, roles, and expectations uniquely influence women’s perceptions and expressions of self, body, overall health, and pain.^13–15^ The collectivist nature of Indian society prioritizes group norms and goals over individual gains and successes.^16^ This thinking has an influence on gender roles, with women most often sacrificing their own wants and needs for the happiness and well-being of their families.^16^ From childhood, young girls are socialized to prioritize others.^15^ In their natal homes, women tend to focus on the needs of their own parents and siblings; after marriage, their priorities shift to their husbands, in-laws, and children.^15^ Married women’s roles largely focus on household responsibilities to maintain loyalty and obedience to the family, such as cleaning and preparing food, performing religious duties, and child-rearing.^17^ This, however, has consequences for their own health and well-being,^15^ as Indian women suffer far worse health consequences than men.^14^

In regards to pain specifically, research shows that Indian women tend to ignore or downplay their pain, as well as restrict its expression in the presence of others, due to cultural beliefs.^15^ Pain is often viewed as God’s will and therefore patience and endurance of pain are valued over open expressions.^18^ One qualitative study exploring the lived experiences of breast cancer symptoms among a group of immigrant Indian women in Canada found that women tended to downplay their pain by attributing it to physical work, rather than viewing it as a sign of greater illness and seeking medical help immediately.^19^ The study also found that when pain became unbearable, women expressed fear of how it may affect their ability to care for their children and husbands.^19^ This further demonstrates women’s prioritization of family members, even when their own health is compromised.

The study of culture is therefore necessary when examining experiences of pain cross-culturally, because beliefs, practices, and norms shape “pain expression, pain language, the cultural context of suffering, traditional healers and lay remedies for pain, [as well as] social roles/expectations. …”^20(p18)^ It is also important to keep in mind the diversity that exists within cultural groups—for example, the many Indian subcultures and religions (i.e., Hindu, Muslim, Christian) that are present—which may influence intracultural variations in pain experiences.

### Immigration

Culture is not static. It shifts and changes with time and place.^21^ Immigration is another key factor influencing pain experiences due to social and cultural changes that occur upon arriving to a new country.^20^ *Acculturation*—“… the extent to which an individual who migrates from the country in which they were born adopts the values, beliefs, culture and lifestyle of their host country”^22(p1,18,6)^—is often a construct used to understand differences in pain experiences among immigrant groups.^23^ A study in the UK, for example, examined the prevalence and severity of pain among Indian, Pakistani, Bangladeshi, and European men and women in relation to immigration and found that acculturation was associated with heightened self-reports of pain. Indian immigrants with the highest acculturation scores also had less widespread pain compared to those with lower acculturation scores.^24^

However, focusing solely on acculturation does not consider other factors associated with the immigration process, such as loss of identity, isolation, structural and economic barriers, and discrimination, which have also shown to have deleterious effects on the health experiences of immigrants in the West.^25^ In particular, changes in housing and working conditions are known to have a negative impact on chronic pain experiences among immigrant populations.^26^ Thus, immigrant status, identity, and structural barriers within host countries^27^ may also influence the experiences of pain for immigrant populations.

### Current Study

Few studies have qualitatively explored the meanings and experiences of chronic pain for immigrant Indian women in Canada.^28^ The current study therefore aimed to determine (1) immigrant Indian women’s lived experiences of chronic pain and (2) the role of culture in these experiences.

## Methods

This research was part of a larger study that employed both semistructured one-on-one interviews and photovoice methods. Only findings from the qualitative interviews are presented here. The study was reviewed and approved by the University of Toronto Research Ethics Board (Approval Number 35563).

### Study Design

We took an interpretive phenomenological approach to explore the lived experiences of chronic pain among immigrant Indian women, particularly drawing on Max van Manen’s^29^ phenomenology of practice. We used this framework because it focuses on lived experiences and the meaning of these experiences as embedded in social, cultural, and historic traditions. This approach calls for a thoughtful and mindful orientation toward the phenomenon and those experiencing it, which allows for an understanding of individuals’ situatedness and “being-in-the-world (p. 38).”^30^ As a methodology, it orients toward a practical approach of “producing action sensitive knowledge.”^31(p21)^ In this context, then, the purpose of understanding lived experiences is to apply it to theoretical knowledge and help inform practice in relation to chronic pain.^32^ In this vein, it can be employed to understand the multiple dimensions of chronic pain from the perspective of the individual’s lifeworld or the “human world of lived experience.”^33(p7,43)^

### Participants

Women were recruited by nonprobabilistic, purposeful sampling (including snowball sampling methods), through the aid of religious centers and nonprofit community organizations tailored specifically for culturally diverse women. These organizations posted recruitment flyers and personally informed women who met the inclusion criteria for the study. If these women showed interest, the lead researcher then contacted them with further questions and details about the study. Once recruited, participants were interviewed at one of the community organizations or within their homes, whichever was most convenient for them.

Inclusion criteria included women who (1) had chronic muscle and joint pain (as defined by pain that has exceeded 3 months)^1^; (2) were between the ages of 40 and 60, plus or minus 3 years; (3) were first-generation immigrants to Canada (people who are born outside of Canada)^34^; (4) had immigrated to Canada from India in the past 10 years (to capture the recent influence of culture and immigration); (5) identified as female; and (6) were able to communicate in English, Hindi, or Urdu. Exclusion criteria included women who (1) had pain associated with cancer; (2) had been in Canada longer than 10 years; (3) were outside the age range; and (4) spoke a language other than English, Hindi, or Urdu. Women of any religion were accepted into the study.

A sample size of six participants is suggested for phenomenological inquiry, because this number is sufficient to produce thick descriptions and interpretations about the phenomenon.^35,36^ However, because of the diversity of Indian women due to various subcultures and religions, we increased the recruitment target to 10 to 15 women.^37,38^

### Interviews

Ten interviews were conducted by the lead investigator in English, two in Hindi, and one in Urdu (no translator was used). Interviews were approximately 1 h and women were asked a number of open-ended questions with respect to their pain experiences; for example, location and duration of their pain, how pain affected their lives, and how they managed pain on a daily basis. In order to further understand the context of their pain experiences, participants were also asked general questions about culture and their experiences of immigration. When these issues naturally arose, women were probed further to explore the influence of the social and cultural context on their experiences of pain. For example, women were specifically asked what cultural beliefs and traditions related to pain and pain management were important to them, as well as how they felt being an Indian woman influenced their experiences of pain.

### Data Analysis

The interviews in English were transcribed verbatim using the qualitative data analysis software QSR International NVivo. For interviews conducted in Hindi or Urdu, a research assistant who spoke these languages (in addition to English) translated and transcribed them into English. The translations and transcriptions were then reviewed in detail by the lead researcher in order to ensure that meaning was accurately captured. A pseudonym that represented their cultural and religious affiliation was also given to each participant corresponding to their participant number.

We used van Manen’s^29^ phenomenological reflection and thematic analysis to analyze the transcripts. Although themes that emerge from phenomenological studies are diverse depending on the research, van Manen^29^ identifies four thematic structures to be reflected on more generally while exploring lived experiences: lived body, lived space, lived human relation, and lived time. A research assistant worked closely with the lead researcher to discuss impressions, observations, and emerging themes from the interviews. Once themes were identified using van Manen’s selective highlighting approach,^29^ they were written in phenomenologically sensitive paragraphs referred to as “linguistic-transformation.”^30(p60)^ The lead researcher also kept a research journal in which she routinely took notes about the women in the study, including observations of participants’ homes and behaviors during interviews, as well as her own reflections.

## Results

No one declined to participate in this study. Thirteen immigrant Indian women participated, and informed written consent was obtained from each of them. The average age of participants was 48 and the average time in Canada was 8 years. These women had immigrated from various regions in India (i.e., Kashmir, Hyderabad, Kerala, and Haryana) to the Greater Toronto Area in Ontario, Canada. They had diverse religious affiliations, including identifications as Hindu, Muslim, and Christian. All women reported chronic musculoskeletal pain and identified pain in knees, legs, hands, or joints. Although some women took over-the-counter pain relief, none of the women were prescribed or were taking prescription pain medication. The average number of years of chronic pain was 7, with some experiencing pain for more than 10 years. Table 1 outlines participant demographics.

**Table 1.** Participant demographics.

Pseudonym	Age	Marital status	Years in Canada	Duration of pain	Location of pain
Diya	49	Married	10	4–5 years	Knees, joints
Ayesha	42	Married	10	2 years	Neck, shoulders, arms
Meera	48	Married	10	5 months	Shoulder, head
Aditi	55	Married	10	4 years	Back, shoulder
Isha	40	Married	2	3 years	Lower limbs, head
Shreya	34	Married	3	6 years	Back
Veda	48	Married	9	7 years	Hands and fingers
Nisha	60	Divorced	10	10+ years	Widespread body pain
Naira	54	Divorced	10	10 years	Widespread body pain
Darsha	36	Married	1	18 years	Legs, shoulders
Maryum	59	Married	10	10 years	Right hand
Shalini	48	Married	5	2 years	Heels, lower back
Sophia	50	Married	10	10+ years	Neck, shoulders, lower back, hands, knees

**Figure 1.**
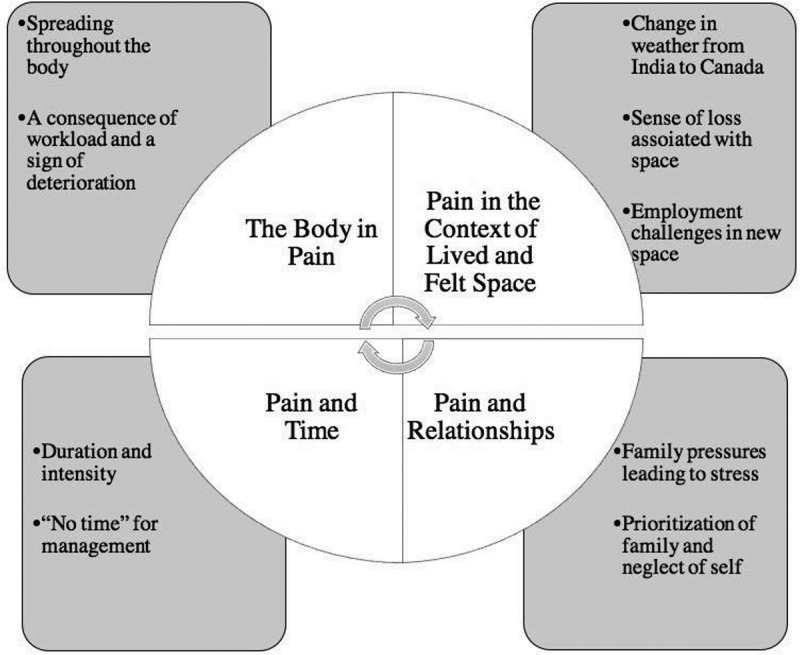
Four main themes and subthemes emerging from interviews.

In line with the four thematic guides of phenomenological analysis as described by van Manen,^29^ emerging themes were grouped under the broad headings of lived experience: (1) the body in pain, (2) pain in the context of lived and felt space, (3) pain and relationships, and (4) pain and time (Figure 1).

### The Body in Pain

Women described pain in their bodies in two ways: (1) as spreading throughout the body and (2) as a consequence of workload and a sign of deterioration.

#### Pain Spreading Throughout the Body

Women spoke of pain beginning in one discrete area and then slowly spreading to other parts of the body. Many described it as starting off localized, in either the neck area, knees, or hands, and then becoming a negative sensation that took over their entire bodies. One participant, Sophia (age 50, 10 years in Canada, English), described it thus: “[The pain] it’s practically all over. It started with my knees but then it’s all over now. My hands, my legs, the joints, the fingers, so you know, every part of my body.”

Bodily restriction and lack of mobility were part of this spreading. Diya said, “… it started from the back and now from the back. … I feel that if I stand up my knees will start hurting, then I feel that my shoulders are freezing” (age 49, 10 years in Canada, English). Naira said, “… pain in my body started and it took over. It spread everywhere, now it’s in my throat, I feel like I can’t breathe at night” (age 54, 10 years in Canada, Hindi).

Women identified that they could no longer move as freely as they once used to because the pain began to affect multiple parts of the body. Diya highlighted that it felt like her “whole body [was] becoming bound.” Therefore, along with multiple body regions of pain sensation, there were also feelings of helplessness, suffocation, and paralysis associated with the pain.

#### Pain as a Consequence of Workload and a Sign of Deterioration

Women spoke a great deal about the physical strains put on their bodies through a never-ending workload since their arrival in Canada. They described a significant increase in the amount of work—both within and outside their homes—required daily since immigrating. Housework—such as errands, taking care of children, cleaning, and cooking for their families—was highlighted as their sole responsibility because they were women. This was exhausting and tiring, as well as harmful to their bodies. Maryum said of her extra workload, “… I had to do everything. Even if it was cold or raining … I had to bring and take the kids, do the work at home, go to the bank, pay bills, go to the doctors, everything I had do to. The struggle was there because I was alone” (age 59, 10 years in Canada, Urdu).

Shalini corroborated this: “[In Canada] sometimes you don’t realize, but you do a lot of work yourself. You do a lot … and you’re always [running] errands doing things and you don’t realize your routine, your body and your life, everything is changing …” (age 40, 2 years in Canada, English).

Pain was also described as a weakening and/or deterioration of the body. For example, Naira described her chronic pain for the past 7 years as feeling like her “bones [were] breaking.” Some associated pain with deficiencies in their body; for example, with a vitamin D or iron deficiency. Some also spoke of their bodies becoming weak after pregnancy and childbirth, which added to, and intensified, the pain they felt. Women felt weak, which was described in terms of no longer being able to do basic day-to-day tasks. For example, Sophia said, “There is muscle and nerve weakness [in my body]. So, my grip … like I cannot carry things. So you know … I was straining my nerves … which over time made my muscles and nerves weak. So, because of all these undetected and untreated ailments … over time it became chronic.”

### Pain in the Context of Lived and Felt Space

#### Change in Weather from India to Canada

Women described their pain in terms of the space surrounding them, particularly in relation to the new spaces, including geography, upon immigrating. Many women attributed the severity of their pain to the change in climate, speaking of having left a warm climate for a cold one. Many said that their pain only began after they moved to Canada, pinpointing the exact beginning of their pain with their immigration. They laid this at the feet of weather. Women highlighted that the cold was not an environment that their bodies were used to. For example, as Diya described, “Climate changes here … you move here from a hot climate place to a cold climate place … what if the cold has settled into your bones?”

#### Sense of Loss Associated with Place

Women expressed the losses of leaving their homeland, one of which was a loss of domestic help. A common discussion throughout the interviews was about having maids back home to help with household tasks, such as cleaning, cooking, and looking after children. This help was easily available to women and accessible at an affordable cost. Having this additional help relieved women of their gendered work—household responsibilities—giving them more time for themselves. Participants were more likely to spend time as they wished back home, were able to pick up hobbies, and had more time to spend with family and friends. This was not so in Canada; the burden of the household chores fell entirely on them after immigration. This played a significant role in the severity of their chronic pain. For example, Sophia stated, “[A] lot of immigrants, I would say, they lose out on or they miss the most on the domestic help which was affordable, you know, outside Canada.”

Moving to Canada and adapting to a new environment also came with the added challenge of losing one’s close social network and cultural familiarity, which meant that they were no longer surrounded by those who understood their gendered roles and values. One woman, Isha (age 40, 3 years in Canada, English), referred to her family and friends in India as a “cushion,” symbolizing comfort and familiarity. She noted that coming to Canada meant that she no longer had this cushion in her life. Immigration and adjusting to a new place caused stress and added to the severity of chronic pain. Isha said, “When we leave everything back home and we just come here wiping off everything … it will increase your stress [and affect] … your physical health also, everything it affects.” Sophia said, “I think initially when I landed, the stress of adjusting, acclimatizing yourself to a new country, new culture … that played I think a very major role in my health.”

There were comparisons between life in India and in Canada in relation to seeking help for pain. Women described trusting the medical system and methods of treatment for pain in India compared to Canada. They spoke of feeling comfortable with, and better understood by, doctors back home and were reluctant to visit medical staff for pain in this new country. Feelings about the Indian care were associated with a sense of belonging, trust, and comfort: “[An Indian doctor will] understand probably because we may be able to connect at that level, but if you are not socially connected to a [doctor], they may not even understand what you are talking about” (Shreya, age 34, 6 years in Canada, English). In addition, Naira said, “Sometimes I think I should go back to India and get treated there, there are good doctors there. Over here, the doctors don’t give you time … you talk to them for 5 minutes, that’s it. What am I supposed to say in that time?”

#### Employment Challenges in New Space

Canadian employment also contributed to women’s pain. Participants highlighted the need to help their husbands financially in order to afford the cost of living in Canada. Though many women were well educated in India—more than half had completed undergraduate degrees from Indian universities and one woman had completed a PhD in botany—almost all complained that their degrees and work experiences were not accepted in Canada and they were therefore forced to work in “survival” jobs (Isha). These were jobs in which women had to work on their feet for long hours; for example, in retail stores, factories, warehouses, and food service jobs. There was talk of how this type of physical labor took a significant toll on their health, not only giving them aches and pains throughout their bodies but—for those who had had pain in India—causing complaints that seemed minor in India to turn major and chronic. Chronic pain in the knees, legs, feet, and hands was commonly discussed in context with this type of work. Isha, for example, discussed how this work was not only difficult but also very different than any type of work she had previously done in India: “So much [stress], we have to push ourselves in warehouses, such kind of jobs, because we haven’t done this in our life before.” Thus, this new place was associated with emotional, resource, and job loss, as well as physically demanding work, all leading to chronic pain.

### Pain and Relationships

Women also spoke a great deal about their pain experiences in the context of their relationships, most often those with their husbands and children. Women identified family stress associated with their responsibilities and duties as another reason pain became chronic in their lives. They also indicated that due to their gender roles, most of their lives were spent prioritizing the needs of others, especially their husbands and children. They felt that they often neglected themselves due to this role and these relationships.

#### Family Pressures Leading to Stress

Women spoke about the expectations and pressures they felt from their families to emotionally and physically support them, even after a long day of tiring work. For example, women were expected to drive children to and from school and extracurricular activities, as well as take care of the elders who often lived with them in a joint family setting. Women felt that these responsibilities, which were associated with their gendered roles and socialization from India, caused them stress in Canada. As Naira noted, “My kids don’t do anything. When I cook, they’ll eat. They’ll maybe wash their plates. Not once have they helped me clean or anything. Cleaning or washing, they don’t do it. … If I am not there, the work will pile up for me.”

#### Prioritization of Family and Neglect of Self

Women focused on their families’ needs to the neglect of themselves and their own health. They explained that even if they were in pain, they tended to ignore it in order to complete their household duties, staying true to their collectivist cultural values. Women indicated that they felt guilty if they were unable to do these tasks because of their pain. Ayesha (age 42, 10 years in Canada, English) said that if she focused on her pain, she would not be able to look after her children: “Because every other day if I am in pain … I wouldn’t be able to take care of kids.”

Neglecting themselves and prioritizing their family were two reasons why women’s pain intensified over the years. They spoke about taking on too much work, thinking that they would be able to handle duties inside the home as well as the pressures of employment outside the home. As Shalini described, “I did not prioritize myself and I overestimated my stamina, my capacity, you can say, to do things.”

Not only were there family expectations but women also placed those very expectations on themselves. They accepted that these were their responsibilities as women, and despite the outside workload adding stress and pain, they continued to carry on because this was culturally what was expected of them. Shreya said: “You always have to cook … so that kind of puts an expectation of course. Coming from a specific ethnic group does affect that, because of how you have been raised.”

### Pain and Time

Pain and time were linked in two ways: (1) duration and intensity of pain and (2) having no time for pain management.

#### Duration and Intensity of Pain

All women described pain as constant and increasing in intensity with time. In fact, the main focus of their health concerns was being in pain all the time, which they found punishing and inescapable. As Ayesha described, “… and with the years, the passing years it got more, more stronger and more I would say, it would last longer … throughout the day, throughout the year … at this point I cannot feel free [of pain] even a minute in 24 hours.”

#### No Time for Pain Management

Women described having almost no time to manage their pain. Although they felt that it would ease their pain to relax, have massages, and rub topical creams on the painful parts of their bodies, due to their busy schedules, their family’s needs, and their work responsibilities, they could not find the time to actually carry out these pain management methods. Though they acknowledged that seeking a doctor’s help was important, as in the case of self-care, there was no time to go to the doctor. Women instead continued their work despite their pain in order to attend to their families, again prioritizing well-being of the collective, rather than themselves. Aditi (age 55, 4 years in Canada, English) stated, “Even if there is pain, still [we Asians] work. And when we are asked to go to the doctor, we don’t get the time.”

## Discussion

Interviews conducted with immigrant Indian women with chronic musculoskeletal pain revealed their lived experiences of chronic pain as multilayered and as having corporeal, spatial, relational, and temporal dimensions. These aspects of pain experiences were heavily tied to their gender roles as Indian women, specifically with respect to the division of household labor. The increased demand to work outside the home also led to changes in gender norms after immigrating to Canada. Both continuing and shifting norms had a significant impact on their experiences of chronic pain.

### Gender, Pain, and the Division of Labor

Women’s narratives centered heavily around their gender role as Indian women. Women’s physical pain experience was described as “bodily restriction” and “paralysis,” echoing gender norms for women in the comportment of their bodies. Their pain narratives were embedded in accounts of family duties. Dube^15^ identified that Indian girls are raised to bear pain, as well as to practice tolerance and self-restraint, which are deeply “… rooted in a consciously cultivated feminine role which is embedded in and legitimized by culture and cultural ideology.”^(p17)^ She, as well as other scholars, highlighted that women in India are raised to display feminine attributes, which dictates the way they walk, talk, sit, and express themselves, and are expected to be docile and maintain a “culture of silence.”^39(p55),40^ All of these gender constraints played into their accounts of pain.

Women were also overwhelmed by work. They spoke of being entirely responsible for the household as well as working outside the home; they identified that this burden of work, which included cleaning, cooking, washing, and running errands, took a significant toll on their bodies. However, even after attributing this workload to their chronic pain, women continued to carry out these chores and described them as mandatory—enacting the gendered division of labor they had in India.^41,42^ In marriage, tasks and duties are gendered.^43^ Women are given the responsibilities of cooking, cleaning, washing clothes, and attending to family members. This “feminine” work is associated with notions of service and sacrifice, implying that it is a woman’s duty to take care of those around her and sacrifice herself.^40,41,44^ These gender norms not only shaped our participants’ chronic pain experiences but also exacerbated them.

### Gender, Pain, and Immigration

Gender and immigration intertwined to affect our participants’ chronic pain experiences. Research has shown that the health of the growing immigrant population in Canada “is primarily affected by dislocation, isolation, loss of identity [and] culture …”^25(p17,14)^ that follows immigration. These factors have been associated with an increase in pain, bodily aches, sleepless nights, and depression, which are found among Canadian immigrant populations.^45^ For the women in our study, the same was true but, in addition, the process of rebuilding and reestablishing their lives in a new country stressed their gender roles, which played out in their chronic pain. This tension affected settlement processes, as women aimed to maintain their traditional, gendered family roles while at the same time dealing with the exigencies of their new environment^21^ and socioeconomic status. The dual roles of homemaker and wage earner increased their pain.

Almost all women in our study noted heavy workloads both inside and outside the home, as well as being homebound by child-rearing and care of elder household members. They associated these burdens not only with taking a physical toll on their bodies but with forcing them to neglect taking care of their pain. In this, our participants are like immigrant Indian women in other studies whose burdens are deemed to have a negative impact on their physical and mental health.^46^ Thus, isolation, heavy domestic workload, and child-rearing responsibilities increase immigrant Indian women’s health problems and chronic conditions, as has been noted for other immigrant women.^47–49^

#### Immigration, New Spaces, and Pain

In addition to the clash of gender roles and increased work burden, women had simultaneous space adjustments; they had to acculturate to a new country and climate as well as find employment in spaces new to them, such as factories and warehouses. Most women who had worked in India were accustomed to jobs in an office or university setting, which did not place a heavy burden on their bodies. In support of women’s experiences from this study, other research has shown greater employment downgrading in both type of work and income for immigrant women compared to men of the same background.^50^ These poor working conditions, alongside traditional gender roles, result in a severe decline in health for particular immigrant groups of women.^51^ This study extends these findings and shows that the additional burden of adjusting to novel demanding work in a new country particularly places immigrant Indian women at risk of severe chronic pain.

As women immigrate from India to Canada, many lose family support and important resources such as paid domestic help.^47^ Almost all women in our study spoke of having had domestic help in India, creating the additional burden of unaided household work after immigration. Previous research has also shown that this extra workload placed on immigrant women from the Indian subcontinent specifically has been linked to a deterioration in their health.^47^ This study therefore extends those findings, showing that this type of housework, coupled with additional stresses of acculturation in a new space, influences women’s experiences of pain.

## Limitations

A strength of our study is that it obtained rich descriptions of immigrant Indian women’s chronic pain experiences. We were, however, unable to restrict our interviews to only one ethnic or religious Indian group. Although all women who participated in the study were born and raised in India before immigrating to Canada, they were from diverse regional and linguistic backgrounds; some were from northern India, and others were born and raised in southern India. Some were Muslim and others were Hindu or Christian. This represents a wide range of cultures with their own gender norms and cultural traditions. Nevertheless, we did find a uniformity of concerns about pain that focused on gender and immigration. Future work would benefit from teasing apart the norms of a given region examining how, and in what ways, they may influence women’s understandings and experiences of pain.

Secondly, although we explored culture in this study in relation to women’s experiences of pain, our aim was not to paint all immigrant Indian women’s experiences with a broad brush. Instead, we wished to explore the influence of cultural context at large on these women’s lives and did so by grounding our findings in women’s personal narratives, stories, and voices. Therefore, the inclusion of non-English-speaking women was a significant strength of the study because a diverse range of pain experiences was captured.

## Implications

The implications of our study for public health systems, clinicians, and health care providers are that Indian women’s cultural values and norms, as well as their daily lives, play a crucial role in their experiences of pain. Their stories are rife with accounts of the tensions they face at the intersection of gender and immigration. Women’s stories can provide information about the triggers of pain, as well as how pain affects their lives, potentially improving both understanding and communication of pain between patients and providers. This, in turn, has the potential to increase culturally responsive pain practices.^52^

In any kind of diagnosis or treatment, it is important to understand the context of the disorder and the patient’s own understandings. It may be, for example, that Indian women will seek health care for what they consider more pressing concerns than pain but, underlying them, they may have chronic pain. Therefore, it may be important to ask about chronic pain directly. Given that the women are serving at least dual roles with little time for themselves and the self-sacrificing gender norms, it is important to consider treatments that may not be seen as time-consuming or selfish. It may also be important to involve other family members as part of the treatment plan.

## Conclusion

Exploring the lived experiences of chronic pain using an interpretive phenomenological approach allowed us to identify the multilayered dimensions of pain experiences for immigrant Indian women in Canada. We found that the lived experiences of chronic pain for women in our study have corporeal, spatial, relational, and temporal components. Our findings reveal that the larger cultural context in which women are raised shape their experiences of chronic pain. Under the umbrella of culture, unique pressures and expectations are placed on immigrant Indian women to continue to fulfill traditional gender roles in the context of immigration challenges. These tensions strongly shaped their experiences of pain. By uncovering the intertwining role of culture, new social structures, gendered lives, and immigrant experiences, we see the complexity of chronic pain and open new doors for therapeutic understanding.
